# Engineered PEG–PCL nanoparticles enable sensitive and selective detection of sodium dodecyl sulfate: a qualitative and quantitative analysis

**DOI:** 10.3762/bjnano.16.29

**Published:** 2025-03-20

**Authors:** Soni Prajapati, Ranjana Singh

**Affiliations:** 1 Department of Biochemistry, King George’s Medical University, Lucknow, India

**Keywords:** Bradford reagent, environmental monitoring, PEG–PCL nanoparticles, SDS, SDS detection

## Abstract

Sodium dodecyl sulfate (SDS) is a widely used anionic surfactant in laboratory, household, and industrial applications, which ultimately enters the environment through various pathways. This has led to significant concerns regarding developing rapid onsite qualitative and quantitative methods for estimating SDS in aqueous solutions. Although a range of high-throughput techniques is currently utilized for SDS quantification, these methods are often expensive, labor-intensive, and require specialized technical expertise. This study developed a novel colorimetric method for the selective and sensitive detection of SDS, utilizing polyethylene glycol-polycaprolactone nanoparticles (PEG–PCL NPs) synthesized via a ring-opening polymerization approach. The synthesized nanoparticles exhibited a distinct colorimetric response to SDS when combined with the Bradford reagent, which acted as a linker molecule. Interference studies demonstrated the high selectivity of the method, even in the presence of various heavy metals and other surfactants. The method showed excellent linearity over a concentration range of 0–200 μg/mL, with a correlation coefficient (*R*^2^) of 0.98. The limits of detection and quantification for the proposed method were determined to be 26.14 μg/mL and 79.23 μg/mL, respectively. These findings indicate that the newly developed method offers high selectivity and sensitivity for SDS detection, making it a promising analytical tool for rapid and onsite estimation.

## Introduction

Sodium dodecyl sulfate (SDS), or sodium lauryl sulfate, is an anionic surfactant widely used in household detergents, personal care products, emulsification, lubrication, catalysis, nanoparticles synthesis, plastic industry, and electroplating [1–4]. This organic compound exhibits an amphiphilic nature, consisting of a 12-carbon hydrocarbon tail covalently bonded to a polar sulfate group, with sodium as the counter-ion. The amphiphilic structure of SDS, integrating a hydrophobic alkyl chain and a hydrophilic headgroup, confers unique physicochemical properties that are leveraged in various applications. However, the widespread use of SDS in different applications led to the need to develop qualitative and quantitative methods for its detection. The SDS estimation is necessary due to its harmful environmental impact, as its use has significantly increased. Exposure to varying concentrations of sodium dodecyl sulfate elicited morphological alterations in the kidney and spleen of the gilthead sea bream (*Sparus aurata L.*), concurrently exerting a significant inhibitory effect on the fertilization process [5]. Twenty juvenile turbot (*Scophthalmus maximus L.*) specimens were exposed to varying concentrations of sodium dodecyl sulfate, which resulted in 50% mortality at 384, 190, 12, and 4 h of exposure, respectively [6]. The sub-lethal chronic effects of SDS were observed on the survival, metabolic processes, and development of juvenile *Centropomus parallelus* specimens exposed to the compound at three distinct salinity levels [7]. In vitro studies on bovine lenses at an SDS concentration of 0.1% to 0.00625% for 30 min showed its toxicity in terms of loss of focus, lens transparency, significant increase in lens wet weight, and axial length. Confocal imaging confirmed concentration-dependent mitochondrial loss in the ocular lens [8]. Human exposure to SDS via air was assessed using the in vitro model system (MucilAir™) at a concentration of ≤10 mM. The study concluded that the release of IL-8 cytokines (≥0.063 mM) increased mucin secretion and decreased transepithelial electrical resistance (TEER) (≥1.25 mM), and the release of lactate dehydrogenase (LDH) (≥2.5 mM) [9].

Apart from that, quantitative and qualitative measurements of SDS in terms of laboratory use are necessary. The SDS is widely used in protein estimation via polyacrylamide gel electrophoresis (PAGE) [10]. Several research groups widely explore nanoparticle synthesis using SDS as a capping agent for different applications [11]. The application of nanoparticles depends on the amount of capping agent adsorbed on the surface of the nanoparticles [12]. This suggests that quantifying the amount of surface capping, such as SDS, is necessary for designing nanoparticle-based applications.

The food industry has been designated as a generally recognized safe (GRAS) ingredient for food applications by the United States Food and Drug Administration (USFDA), as stipulated in 21 CFR 172.822. This anionic surfactant serves dual functions in food processing: as an emulsifying agent and a whipping aid [13]. The United States Code of Federal Regulations has established specific concentration limits for SDS in various food products. When utilized as an emulsifier in conjunction with egg whites, the permissible maximum concentration is 1,000 parts per million (ppm) or 0.1% in egg white solids and 125 ppm or 0.0125% in frozen or liquid egg whites. In the context of marshmallow production, where SDS functions as a whipping agent, its concentration must not exceed 0.5% of the gelatine weight. Interestingly, SDS has been reported to exhibit a temporary suppressive effect on the perception of sweetness, which warrants further investigation in food science and sensory analysis. SDS is extensively utilized in formulating oral hygiene products, owing to its cost-effectiveness and efficacious properties as a foaming agent [14].

The above studies confirmed the role of SDS and its widespread use in different applications within permissible limits. To assess the safe dose of SDS in other products, various high throughput tools are available, including spectrophotometrics, potentiometrics, high-performance liquid chromatography (HPLC), capillary electrophoresis, and fluorescence-based methods [15–19]. The techniques mentioned are expensive, use toxic chemicals, are laborious, and require highly skilled professionals. Apart from that, each method has limitations, such as chromatographic techniques requiring the separation of the target analyte from complex mixtures. The commonly employed ionometric technique for quantifying surfactants employs ionic electrodes to measure the unknown concentration of the target substance within 30 minutes. However, this method exhibits reduced sensitivity (280–600 µg/mL) and selectivity, rendering it less than optimal for analyzing surfactants in relatively complex matrices [20]. Spectrophotometric techniques offer a straightforward approach and demonstrate a high level of sensitivity, capable of detecting concentrations as low as 0.001 μg/mL [21–22]. The spectrophotometric technique drawbacks encompass reduced specificity and the need for sample dilution to fall within the 0.01 μg/mL measurement range, rendering the process cumbersome [21]. Also, an instrument is required among all the reported techniques, making detecting SDS at a target site complicated for the non-technical person. Therefore, there is an urge to develop a simple, colorimetric, rapid, nontoxic, selective, and cost-effective sensor for SDS detection in an aqueous solution.

The advent of nanotechnology increased their demand for developing colorimetric sensors to detect chemical compounds, toxicants, nutrients, and biomolecules. The nanotechnology field consists of synthesis, characterization, and manipulation of particles with a size less than 100 nm. These tiny particles possess unique physicochemical features, including optical, electrical, magnetic, and catalytic properties [23]. Indeed, the advanced properties of nanoparticles enables them to be used in different areas, such as biosensing, drug delivery, targeting, sensing, and imaging [23]. Also, there are a wide variety of nanoparticles available for desired applications. In the case of detection of contaminants or sensing applications, carbon and metal nanoparticles are mostly preferable [24]. Despite their use, these nanoparticles possess inherent toxicity, and their post-synthesis functionalization is laborious for selective detection [25–26].

Furthermore, alternatives such as polymer nanoparticles emerged as potential candidates for detecting contaminants such as SDS. Fu et al. developed a method for detecting SDS using polyethyleneimine (PEI) and ascorbic acid [27]. However, their approach is fluorescence-based, which makes it difficult to estimate at the target site and also requires a sophisticated instrument such as a spectrofluorometer. Consequently, developing a simple colorimetric detection method for SDS using polymer nanoparticles is a step ahead of the reported studies. Polymer nanoparticles prepared from PEG–PCL are widely used for drug delivery, tumour targeting, and imaging [28]. To our knowledge, there is no report regarding using PEG–PCL nanoparticles (PEG–PCL NPs) as a contaminant detection system. Accordingly, we hypothesize that PEG–PCL NPs can serve as effective colorimetric probes for SDS detection, addressing the need for a rapid, nontoxic, selective, and cost-effective environmental and human health protection sensor. The current study is based on developing a colorimetric/spectrometric sensing probe for SDS using PEG–PCL NPs. Thus, this study is the first of its kind to develop a colorimetric/spectrometric detection system for anionic surfactants.

## Materials and Methods

### Materials

Methoxy polyethylene glycol (mPEG5000, Cat. No. 81323), ε-caprolactone (Cat. No. 704067), stannous octoate (Cat. No. S3252), cetyltrimethylammonium bromide (CTAB, Cat. No. H5882), sodium dodecyl sulfate (SDS, Cat. No. 7910), Tween 20 (Cat. No. P1379), and Triton X-100 (Cat. No. T8787) were acquired from Sigma-Aldrich (USA). Dichloromethane (DCM, Cat. No. 24532), diethyl ether (Cat. No. 64665), phosphate-buffered saline (PBS, Cat. No. 78529) and Bradford reagent (Cat. No. 19219) were purchased from SRL Chemicals (India). NIST-grade standards of arsenic (As^3+^), aluminium (Al^3+^), cadmium (Cd^2+^), zinc (Zn^2+^), mercury (Hg^2+^), nickel (Ni^2+^), copper (Cu^2+^), chromium (Cr^3+^), lead (Pb^2+^), iron (Fe^3+^), and cobalt (Co^2+^) (Cat. No. 041865), as well as silicon oil (Cat. No. 015067), were obtained from CDH Fine Chemicals (India). All chemicals were used as received without further purification. Prior to experimentation, all glassware was cleaned with aqua regia (HCl:HNO_3_, 3:1 v/v) and thoroughly rinsed with double-distilled water.

### Methodology

#### Synthesis of PEG–PCL nanoparticles

PEG–PCL copolymer nanoparticles were synthesized via a modified ring-opening copolymerization method with slight modification using a previously reported procedure [29]. In a typical synthesis, 4 g of mPEG was added to a round-bottom flask purged with nitrogen gas and vigorously stirred in a silicon oil bath at 130 °C. Subsequently, a syringe introduced 2 mL of ε-caprolactone and 1 mL of stannous octoate (as a reaction catalyst) into the molten mPEG. The polymerization reaction was conducted under vacuum with continuous stirring at 130 °C for 24 h. After the polymerization, the resulting complexes were cooled down to room temperature, dissolved in DCM, and precipitated using an excess of cold diethyl ether. The precipitates were isolated by filtration using filter paper and dried under vacuum. The collected PEG–PCL NPs were then subjected to further characterization.

#### Characterization of PEG–PCL nanoparticles

The synthesized PEG–PCL nanoparticles were characterized by their unique physicochemical properties, such as size and surface charge. The average hydrodynamic size, monodispersity, and surface charge of the nanoparticles were measured using the ZetaSizer (Nano ZS, Malvern, UK). Dried 10 mg/mL PEG–PCL NPs were dissolved in PBS and sonicated for 30 min. The sonicated sample was taken in a cuvette for measurement. The sample pH was in the range of 7.2–7.4. Separate cuvettes (DTS1072 and DTS0012) were used to measure the surface charge and average size of the nanoparticles. Polystyrene latex absorption coefficient and refractive index were used to measure synthesized nanoparticles, prefilled in the software with values of 0.01 and 1.59, respectively. All measurements were performed at 25 °C. The surface morphology of PEG–PCL nanoparticles was analyzed using scanning electron microscopy (SEM). The nanoparticles obtained from the 10 mg/mL stock solution were diluted 100-fold for the SEM analysis. The slide for SEM imaging was prepared with a sputter coating of gold as a conductive material, followed by the addition of 10 μL of nanoparticles, and air drying. The imaging was performed using SEM (FEI Quanta 250, Netherlands). Transmission electron microscopy (TEM) was also performed to measure nanoparticle mean size and their distribution. The sample was diluted 1000-fold from the stock solution, and 5 µL of the sample was placed onto a carbon-coated copper grid with 200 mesh size. The imaging was performed using TEM at 120 kV (Jeol JEM1400, Germany). The surface elements and their composition in the nanoparticle were analyzed using X-ray photoelectron spectroscopy (PHI 5000 Versa Probe II, FEI Inc) regarding their binding energy. The fixed transmission mode was utilized with passing energy at 80 eV, and the binding energy spectrum was recorded from 0 to 1,400 eV. The functional group interaction of PEG–PCL nanoparticles was assessed using Fourier-transform infrared (FTIR) spectroscopy (Thermo Scientific, Nicolet 6700). An amout of 5 mg of PEG, PCL, and PEG–PCL nanoparticles was placed over the diamond point, and the spectra were recorded in the 3900–550 cm^−1^ range.

#### Optimization of PEG–PCL nanoparticles

The concentration and volume of PEG–PCL NPs were optimized using Bradford reagent to obtain maximum absorbance. PEG–PCL NPs (10 mg/mL) were added to the Bradford reagent, and spectrophotometric absorbance was measured in different ratios. Finally, different concentrations of PEG–PCL NPs (10–0.005 mg/mL) were added to the Bradford reagent, and colorimetric change was examined.

#### Qualitative detection of sodium dodecyl sulfate using PEG–PCL nanoparticles

The qualitative SDS detection was performed using colorimetric and spectrophotometric approaches. For the detection experiment, a range of commonly employed surfactants representing different classes: cationic (CTAB), anionic (SDS), and nonionic (Tween 20 and Triton X-100) was tested at a final concentration of 0.1% (w/v). In order to make sensitive detection of SDS, various metals such as Al^3+^, Cd^2+^, Zn^2+^, Hg^2+^, Ni^2+^, Cu^2+^, Cr^3+^, Pb^2+^, Fe^3+^, Co^2+^, and As^3+^, each at a final concentration of 1 ppm were included in the study. The reaction mixture consisted of 400 μL of polyethylene glycol-polycaprolactone (PEG–PCL) nanoparticles (10 mg/mL), 500 μL of Bradford reagent, and 100 μL of the respective metal ion solution or surfactant, with a total reaction volume of 1 mL. Visual color changes were observed, and absorbance was measured in the 300–900 nm range using a spectrophotometer (Multiskan G0, Thermo Scientific, USA).

#### Interference and quantitative analysis

The specificity of SDS detection was evaluated through interference studies in the presence of various metal ions and surfactants. Three experimental groups were established: (1) a control group without added surfactants or metals, (2) a comprehensive group containing all tested metals and surfactants, including SDS designated as SDS (+), and (3) a group encompassing all surfactants and metals except SDS designated as SDS (−). Colorimetric and spectrophotometric analyses were conducted to assess the response in each group. The quantitative estimation of SDS was calculated using a linear equation between the concentration of SDS and their respective optical density. For a quantitative estimation of SDS, a series of samples with decreasing SDS concentrations were prepared to determine the detection limit while maintaining constant concentrations of PEG–PCL NPs and the Bradford reagent. The colorimetric changes were visually observed, and the corresponding spectral absorbance was measured using spectrophotometry.

## Results and Discussion

### Synthesis of PEG–PCL nanoparticles

**Figure 1:**
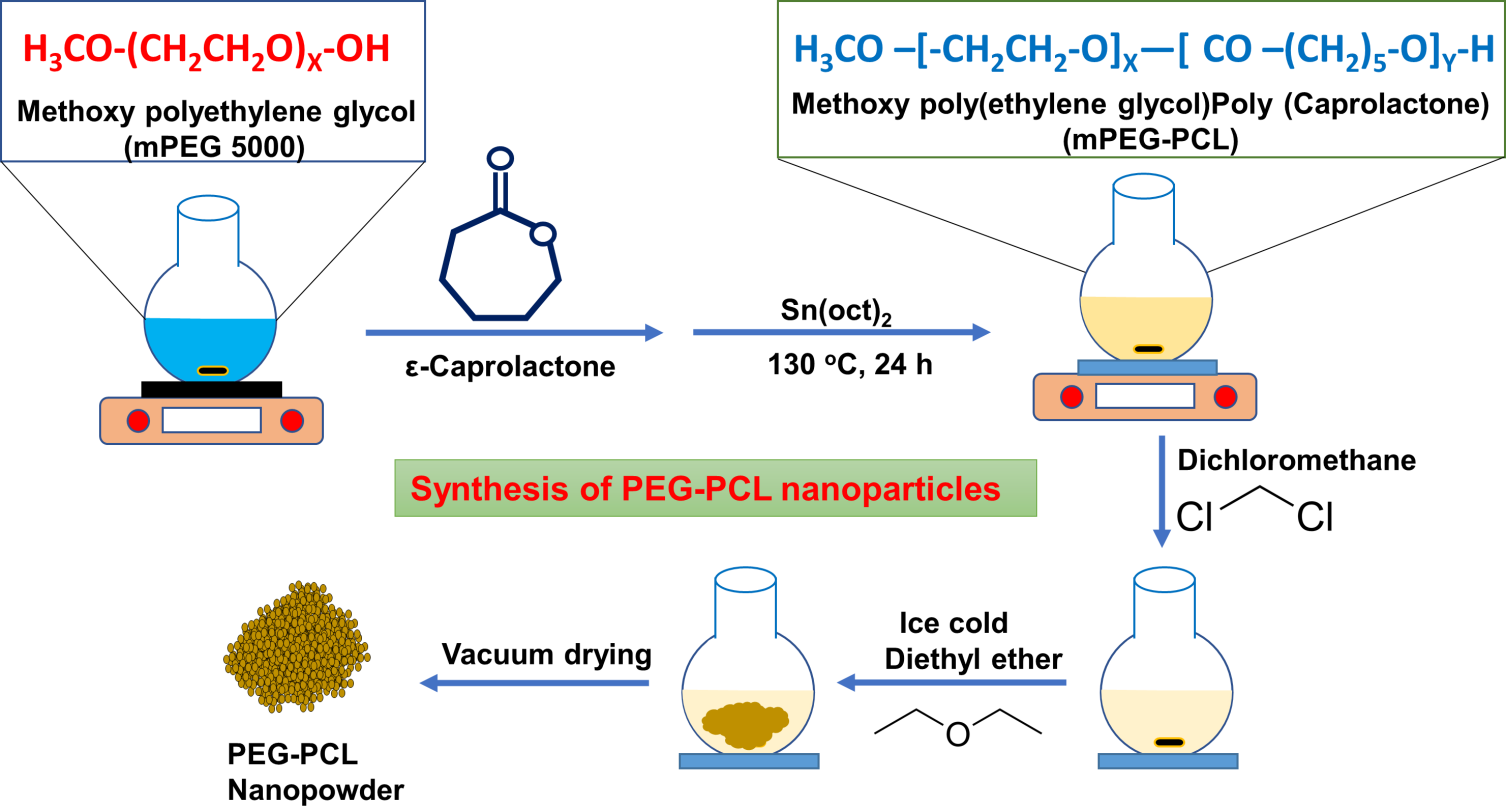
Schematic representation of PEG–PCL nanoparticle synthesis via ring-opening copolymerization (ROC). The diagram illustrates the stepwise chemical process of nanoparticle formation, highlighting key molecular transformations and reaction conditions involved in the polymerization method.

The synthesis procedure of PEG–PCL nanoparticles and their interaction mechanism are discussed in Figure 1.

Polyethylene glycol–polycaprolactone nanoparticles were synthesized through ring-opening copolymerization (ROC), a widely recognized technique for creating nanoparticles with tailored properties (Figure 1). This method leverages the complementary characteristics of PEG and PCL to produce amphiphilic nanoparticles which possess both hydrophilic and hydrophobic segments. PEG, known for its water solubility and biocompatibility, provides the hydrophilic component, while PCL, a biodegradable polyester, contributes with hydrophobicity, enabling the formation of nanoparticles that can interact with both aqueous and non-aqueous environments. The synthesis begins with the interaction of the metal oxide initiator, tin(II) 2-ethylhexanoate (Sn(oct)_2_), with the monomers ethylene oxide and ε-caprolactone. Sn(oct)_2_ acts as a catalyst, activating the monomers by generating reactive metal oxide species. These reactive species facilitate the ring-opening polymerization of ethylene oxide and ε-caprolactone, initiating copolymerization. During this process, the monomers alternately add to the growing copolymer chain, forming blocks of distinct PEG and PCL segments. This alternating addition is crucial for creating the amphiphilic structure, where PEG provides the hydrophilic domains, and PCL forms the hydrophobic domains. The copolymerization reaction can proceed for approximately 24 hours, during which nucleation and growth of the nanoparticles occur. The reaction duration ensures sufficient time for the formation of well-defined, monodisperse nanoparticles with consistent size and shape. The monodispersity of the nanoparticles is critical for their uniform behavior in biological and industrial applications, as it influences factors such as drug loading efficiency and release kinetics. After copolymerization, the reaction is terminated by dissolving the resulting copolymer in DCM, a solvent that allows the copolymer to remain in solution. The solution is then precipitated in cold diethyl ether, which helps to remove unreacted monomers and other impurities. This precipitation step is essential for purifying the nanoparticles and achieving a stable, solid-state product that can be easily collected and dried. Finally, the synthesized PEG–PCL NPs are characterized to assess their size and uniformity. Dynamic light scattering (DLS) techniques measure the average particle size and polydispersity index (PDI). These characteristics are crucial for ensuring the reliability and reproducibility of the nanoparticles in various applications, including sensing.

### Characterization of PEG–PCL nanoparticles

**Figure 2:**
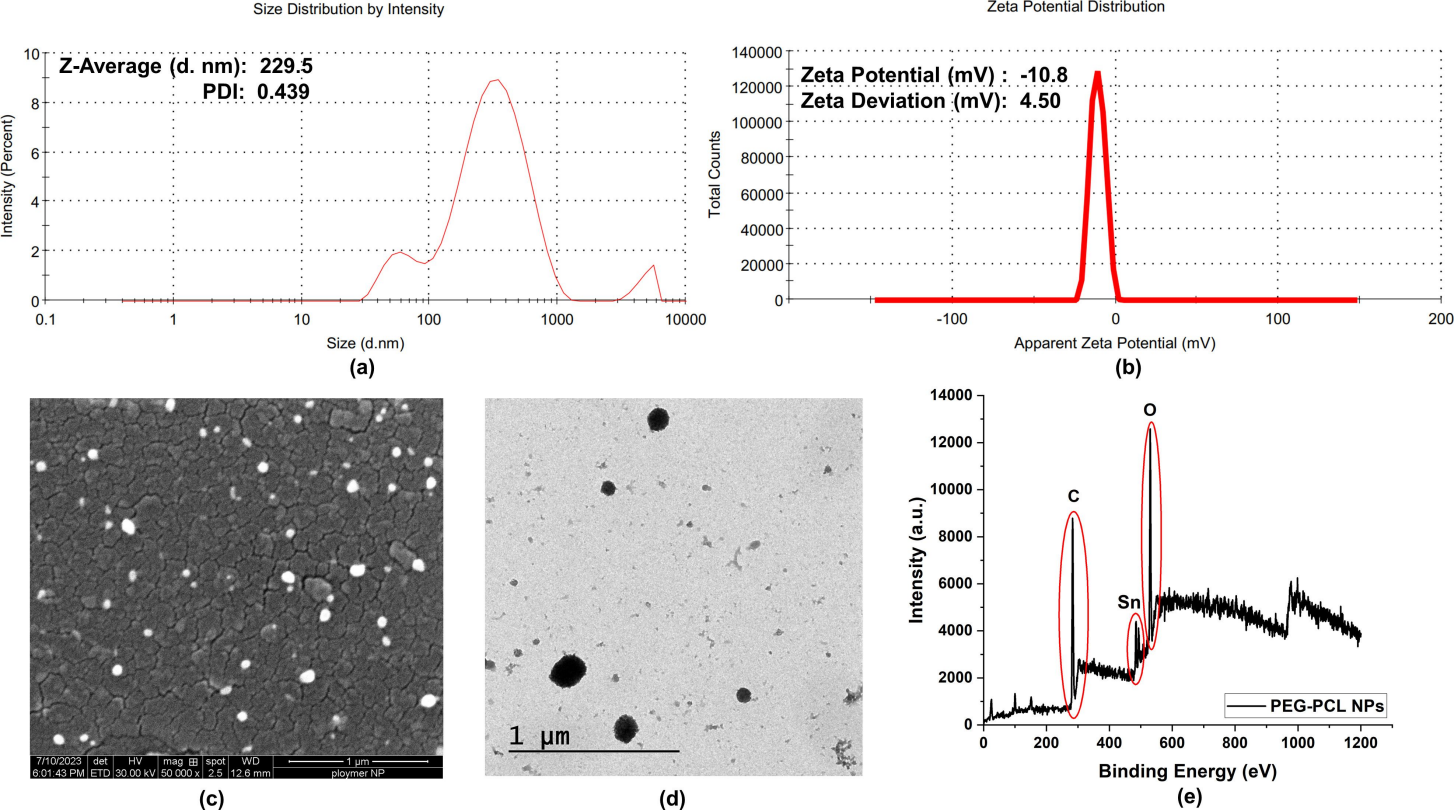
Physicochemical characterization of PEG–PCL nanoparticles: a) hydrodynamic radius and PDI, b) zeta potential, c) SEM, d) TEM, and e) XPS analysis.

The prepared PEG–PCL nanoparticles were characterized for their physicochemical properties, such as size, shape, surface charge, and elemental composition using DLS, TEM, SEM, and XPS (Figure 2).

The PEG–PCL NPs were characterized using a zetasizer (Nano ZS, Malvern, UK) to comprehensively assess their size, surface charge, monodispersity, and average hydrodynamic size. Dynamic light scattering measurements revealed that the average hydrodynamic radius of the PEG–PCL NPs was 229.5 nm, confirming their nanoscale dimensions. This finding aligns with previously reported studies that detail the properties of PEG–PCL NPs in similar applications [29]. The polydispersity index, a measure of molecular mass distribution in a given polymer sample, was calculated to be 0.439 (Figure 2a). Typically, a PDI value near 0.3 indicates a monodisperse particle distribution, which is desirable for uniformity in size and performance [30]. However, the slightly elevated PDI observed here suggests a small degree of polydispersity, indicating that while the nanoparticles are relatively uniform, there is a slight variation in their sizes. The zeta potential of the PEG–PCL nanoparticles was measured to be −10.8 ± 4.50 mV (Figure 2b), suggesting that the nanoparticles possess a moderate negative surface charge. The zeta potential is a critical parameter for evaluating the stability of colloidal dispersions; typically, values greater than ±30 mV are associated with high stability due to strong electrostatic repulsion between particles [31]. Despite the zeta potential being less than ±30 mV, the negative charge still provides sufficient electrostatic repulsion to minimize aggregation. This repulsion is key to maintaining a stable colloidal suspension, as it prevents the nanoparticles from clumping together, which is crucial for their effective use in various applications, such as drug delivery and biosensing. Further insights into the size and morphology of the PEG–PCL NPs were obtained through electron microscopy. Scanning electron microscopy was used to examine the surface structure and to conduct a quantitative size distribution analysis. The SEM images (Figure 2c) revealed that the PEG–PCL NPs have smooth and homogenous surfaces with small pore sizes. The nanoparticles demonstrated high uniformity and were predominantly quasi-spherical in shape, with an average size of 53.7 ± 10 nm. This quasi-spherical morphology is advantageous for many applications, as it provides a high surface-area-to-volume ratio, which enhances interaction with target molecules. Transmission electron microscopy was also employed further to confirm the shape and size of the nanoparticles. The TEM images confirmed that nanoparticles were spherical with a narrow size distribution, and the mean size was 48.3 ± 16.4 nm (Figure 2d). The slight discrepancy in size measurements between TEM, SEM, and DLS can be attributed to the different operational principles of these techniques. DLS measures the hydrodynamic diameter, including the particle core and the layer of solvent molecules attached, leading to a larger estimate than the dry measurements obtained from TEM and SEM [32]. X-ray photoelectron spectroscopy, also known as electron spectroscopy for chemical analysis (ESCA), was used to analyze the surface chemistry of the PEG–PCL NPs. XPS is a powerful surface-sensitive technique that provides detailed information about the elemental composition, chemical states, and electronic states of the elements present in the nanoparticles. The XPS spectra of the PEG–PCL nanoparticles (Figure 2e) showed prominent peaks at binding energies of 284.8, 532.7, and 486.7 eV, corresponding to carbon, oxygen, and tin, respectively. The presence of carbon and oxygen peaks confirms the composition of the PEG–PCL polymer matrix, while the tin peak is attributable to the stannous octoate catalyst used during the synthesis of the nanoparticles. The absence of unexpected peaks in the XPS spectra indicates that no significant elemental changes occurred during the synthesis process, suggesting the stability and integrity of the PEG–PCL NPs. This consistency in elemental composition further supports the use of these nanoparticles in various applications, as it ensures that the nanoparticles retain their designed properties without undergoing undesirable chemical changes.

**Figure 3:**
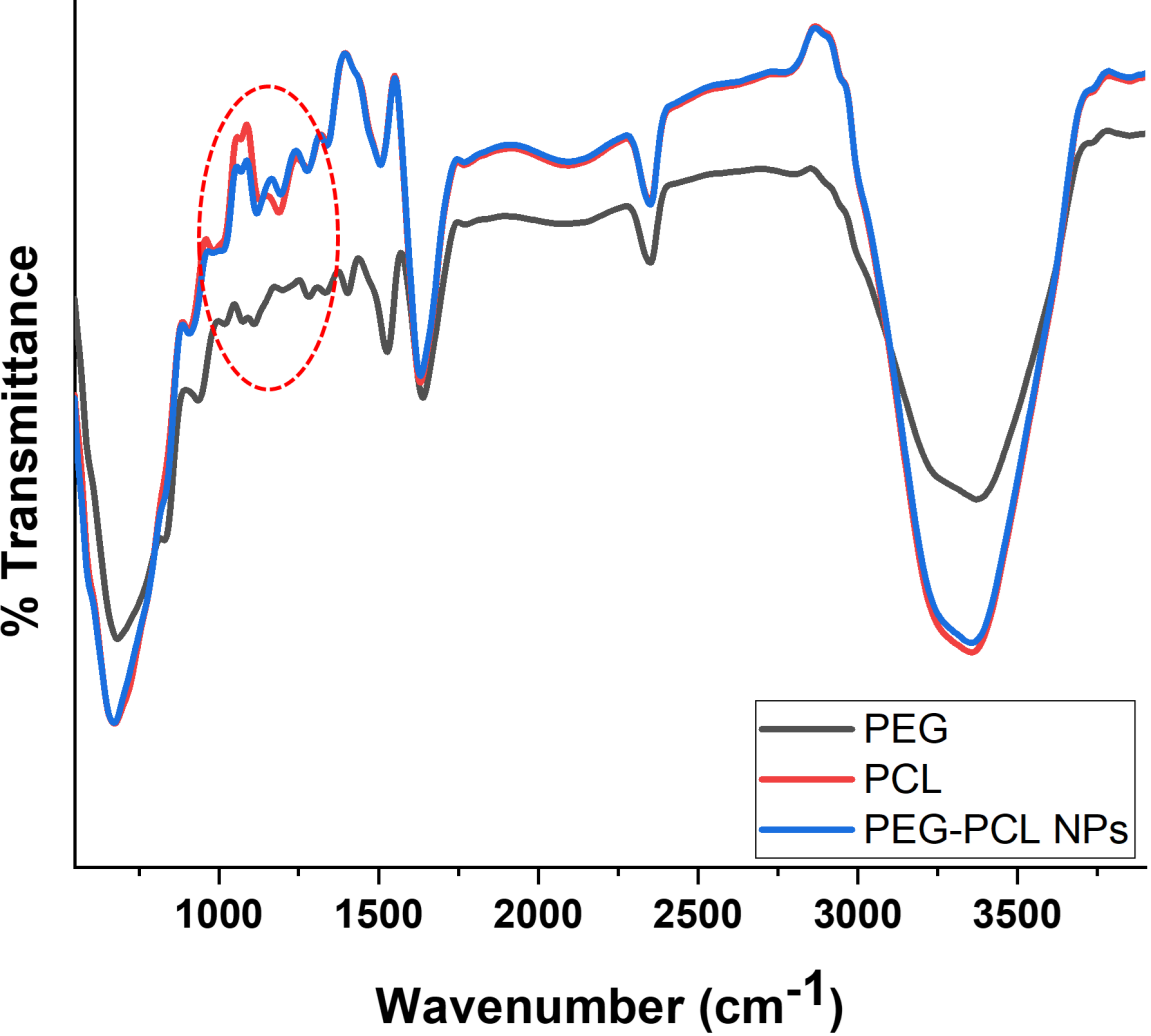
FTIR spectrum of PEG–PCL nanoparticles showing functional group interaction between parent compounds (PEG and PCL).

The functional group interactions between PEG and PCL to form PEG–PCL nanoparticles were investigated using FTIR spectroscopy (Figure 3).

The FTIR spectrum of PEG–PCL nanoparticles reveals several characteristic peaks indicating their chemical structure and successful synthesis (Figure 3). The broad peak at 3357 cm^−1^ corresponds to the O–H stretching vibrations, typically from the terminal hydroxyl groups of PEG segments. The peaks at 2790 cm^−1^ can be attributed to the symmetric and asymmetric C–H stretching vibrations of methylene (–CH_2_–) groups present in both PEG and PCL segments. The absorption band at 1628 cm^−1^ represents the characteristic C=O stretching vibration of the ester groups in PCL blocks. The peak at 1510 cm^−1^ can be assigned to the C–H bending vibrations of the methylene groups. The peaks at 1336 and 1273 cm^−1^ correspond to the C–O and C–C stretching in the crystalline phases of PCL. The bands at 1187 and 1119 cm^−1^ are attributed to C–O–C stretching vibrations, characteristic of the ether linkage in PEG and the ester groups in PCL. The 1019 and 905 cm^−1^ peaks can be assigned to the C–O stretching and C–H rocking vibrations, respectively. The presence of peaks at 2349 and 2073 cm^−1^ might be due to ambient CO_2_ absorption or other environmental factors during measurement. This spectral analysis confirms the successful incorporation of PEG and PCL into the nanoparticle structure, showing their characteristic functional groups and molecular interactions. Previous studies confirmed that PEG contains hydroxyl groups (–OH) that can form hydrogen bonds with the PCL carbonyl groups (C=O). These hydrogen bonds help stabilize the nanocomposite structure and improve its mechanical properties [33].

### Optimization of PEG–PCL nanoparticle concentration

**Figure 4:**
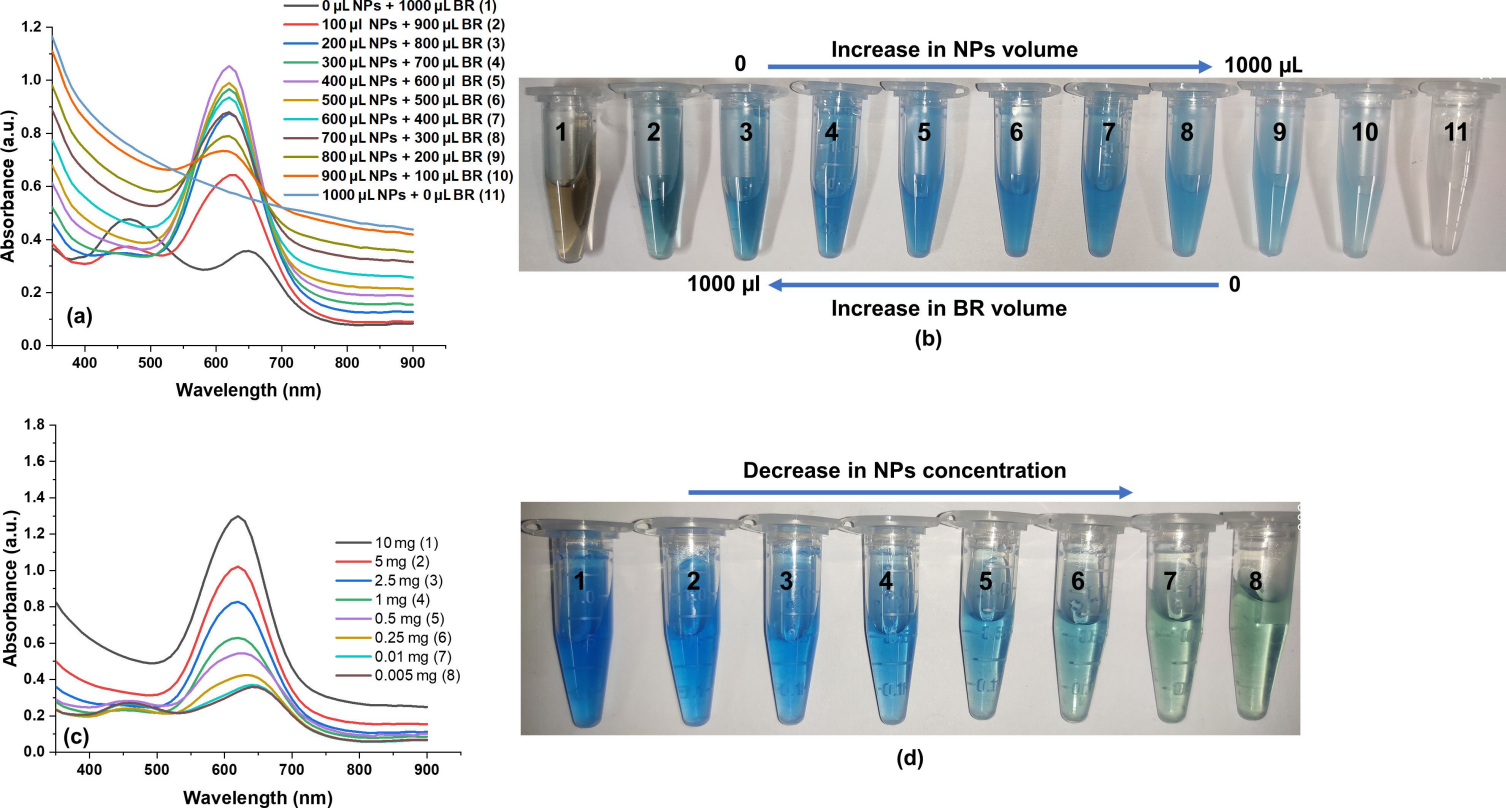
Optimization of PEG–PCL nanoparticle concentration. a) Spectrophotometric absorbance and b) colorimetric change of different ratios of PEG–PCL NPs and the Bradford reagent (BR). c,d) Spectrophotometric absorbance and colorimetric change of different concentrations of PEG–PCL nanoparticles in a fixed Bradford reagent, respectively.

The PEG–PCL NP concentration was optimized for optical absorbance in the presence of the Bradford reagent for SDS detection. As shown in Figure 4a, the optical absorbance of the Bradford reagent was significantly increased upon increasing the volume of PEG–PCL nanoparticles (10 mg/mL) from 0–1000 μL. However, extreme values such as 0 and 1000 μL showed no significant difference in plasmonic absorbance. This suggests that without PEG–PCL nanoparticles (i.e. 0 μL), only Bradford reagents cannot produce substantial optical absorbance.

Similarly, in PEG–PCL NPs, no absorption band was observed in the absence of the Bradford reagent. This confirmed that both PEG–PCL NPs and the Bradford reagents need to be added to the ratio for the optical sensor development. PEG–PCL NPs and the Bradford reagent ratio was 2:3, giving maximum optical absorbance (Figure 4a). Similar results were obtained via colorimetric observation through the naked eye, as shown in Figure 4b. However, the Bradford reagent inherently produces a brown color, which is changed to a blue color upon adding different volumes of PEG–PCL NPs. The PEG–PCL nanoparticles are colorless and attain color only after adding the Bradford reagent in a particular ratio. Coomassie Brilliant Blue G-250 is the main component of the Bradford reagent. This suggests that PEG–PCL NPs and Bradford reagents work synergistically but not independently. The G-250 was adsorbed over the surface of the nanoparticles, composed of hydrophilic PEG and hydrophobic PCL [34]. The positive charge on the G-250 dye or the Bradford reagent can form ionic interactions with PEG–PCL NPs due to their negative surface charge, which is confirmed through the zeta potential. We also observed when the PEG–PCL nanoparticle concentrations decreased from 10 to 0.005 mg/mL, the plasmon peak significantly decreased due to a lower number of nanoparticles, and their corresponding color significantly changed compared to the control value. This suggests that nanoparticle concentration plays a significant role in determining the nanoparticle–Bradford reagent interaction for detection of SDS (Figure 4c,d).

### Selective colorimetric/spectrophotometric detection of SDS using PEG–PCL NPs

**Figure 5:**
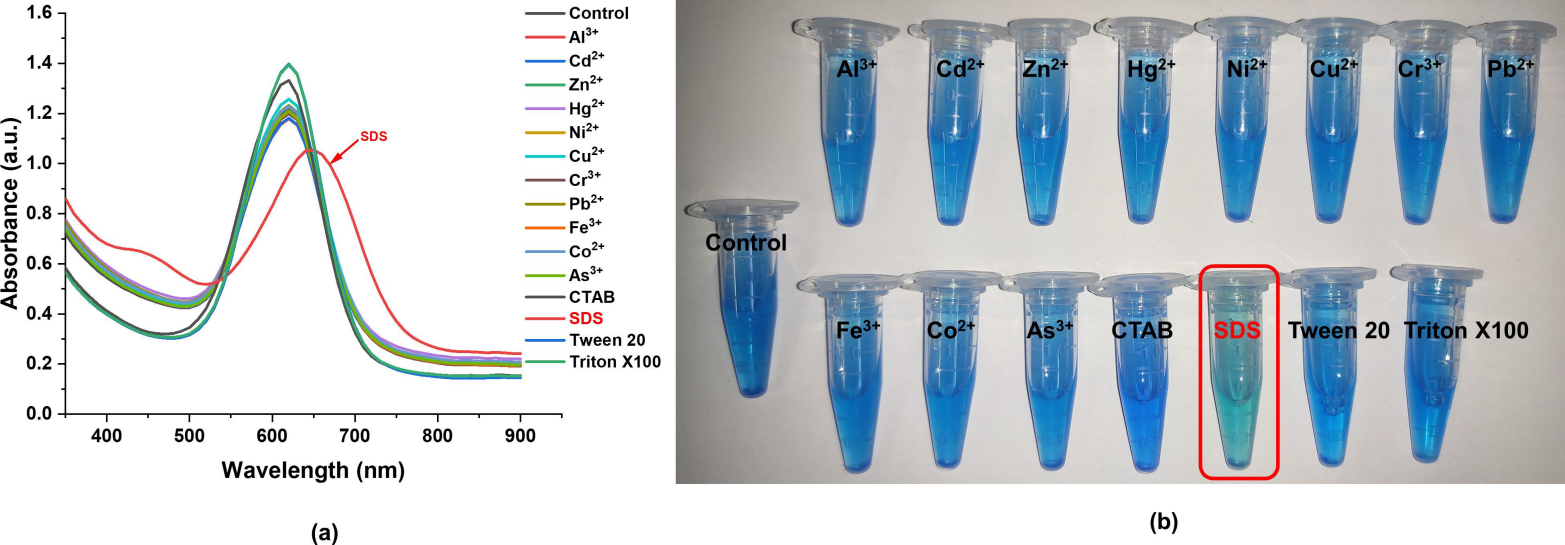
Selective a) spectrophotometric and b) colorimetric detection of SDS using PEG–PCL nanoparticles among different metal ions and surfactants. The red marking denotes detected SDS among other samples.

The synthesized PEG–PCL nanoparticles demonstrated a unique colorimetric response in the presence of the Bradford reagent. This property was exploited to detect SDS using colorimetric and spectrophotometric methods. Specifically, when combined with the Bradford reagent, the PEG–PCL nanoparticles produced a distinct blue color, indicating a successful interaction. This interaction generated a sharp plasmon resonance peak with a maximum absorbance (λ_max_) at 620 nm. Further, the addition of SDS to PEG–PCL nanoparticles showed a redshift of 30 nm in plasmonic absorbance, indicating the specific interaction between the nanoparticles and SDS in the presence of the Bradford reagent (Figure 5a). This suggests that only SDS is actively involved in nanoparticle interaction with a measurable optical change that can be spectrophotometrically quantified. To assess the selectivity and specificity of the detection system towards SDS, a range of heavy metal ions (including Al^3+^, Cd^2+^, Zn^2+^, Hg^2+^, Ni^2+^, Cu^2+^, Cr^3+^, Pb^2+^, Fe^3+^, Co^2+^, and As^3+^) at a concentration of 1 ppm were introduced, along with various surfactants such as CTAB, SDS, Tween 20, and Triton X-100 at a concentration of 0.1%. These ions and surfactants were chosen due to their relevance in environmental samples, and are example of common pollutants. The study found that while these heavy metal ions and other surfactants were present, the PEG–PCL nanoparticles showed a selective response to SDS. Specifically, only in the presence of SDS a significant redshift of approximately 30 nm was observed in the plasmon resonance peak. This redshifting of absorbance maximum to a longer wavelength is a unique response not seen with the other tested ions or surfactants (Figure 5a). This selective shift indicates a specific interaction between SDS molecules and the PEG–PCL nanoparticles, facilitated by the Bradford reagent, which acts as a linker, enhancing the sensitivity of the system. Furthermore, this spectrophotometric shift corresponded to a visible color change from deep blue to lighter blue, which could be easily discerned by the naked eye (Figure 5b). The ability to detect this color change within a short time frame, within five minutes of initiating the reaction, demonstrates the rapid response of the system, making it highly suitable for quick onsite SDS detection. This rapid colorimetric response, coupled with the specificity and selectivity of the system, highlights the potential of the PEG–PCL nanoparticle-based method as a robust tool for environmental monitoring and analysis of SDS contamination. The simplicity of visual detection, alongside quantitative spectrophotometric measurements, provides a dual approach that can cater to different levels of detection requirements in various practical applications.

### Proposed mechanism for SDS detection

Ionic charge interactions between SDS and the Bradford reagent primarily drive the detection mechanism for SDS using PEG–PCL NPs [35–36]. SDS is an anionic surfactant with a negative charge, while the Coomassie Brilliant Blue G-250 dye in the Bradford reagent carries a positive charge. In addition, PEG–PCL NPs adsorbed the Bradford reagent and produced a plasmonic peak. The absorbance peak in PEG–PCL NPs is due to the interaction with the Bradford reagent, which is further used for the detection of SDS via electrostatic interaction. An ionic interaction occurs when the negatively charged SDS molecules come into contact with the PEG–PCL absorbed with positively charged G-250 dye (from the Bradford reagent). This interaction disrupts the local environment around the dye molecules, changing the optical properties of the PEG–PCL NPs–Bradford reagent complex. The ionic interactions cause the G-250 dye (from the Bradford reagent), which originally exhibits a brownish color, to change into a blue color upon binding to the PEG–PCL NPs. As such, PEG–PCL NPs are colorless. Adding SDS further modifies this interaction, resulting in a red shift from blue to light blue. This color change is a direct consequence of the formation of the SDS–PEG–PCL NPs–dye complex, which alters the electronic structure of the dye molecules and leads to a shift in their absorption characteristics. The resultant light blue color in the presence of SDS can be easily detected and quantified using spectrophotometric techniques, providing a straightforward method for SDS detection. Previous research has established that SDS can interact with the G-250 dye in the Bradford method, a commonly used method for protein quantification [36]. However, the interaction is not visually or optically measured without protein in a sample. In typical protein quantification methods, the interaction of the G-250 dye with proteins produces a plasmon resonance peak around 595 nm, forming a blue form of the dye–protein complex [37]. Furthermore, the quantification of SDS is not possible with a dye, only without involving protein. Therefore, the current study utilized NPs with PEG–PCL to detect SDS in the presence of the Bradford reagent or G250 dye, making the process easy and cost-effective. The PEG–PCL NPs–dye complex exhibits an absorbance peak at 620 nm when mixed with the Bradford reagent. This shift in the plasmon peak suggests that PEG–PCL nanoparticles, in conjunction with the G-250 dye (from the Bradford reagent), may mimic protein-like behavior, promoting a more stable interaction and color change from brown (Bradford reagent) to blue (PEG–PCL NPs–Bradford complex). Furthermore, when SDS is introduced into the system containing PEG–PCL NPs and Bradford reagents, the color changes from blue to light blue along with a prominent plasmonic shift. This shift indicates a unique and selective interaction between SDS molecules and the nanoparticle–dye complex. The shift from 620 to 650 nm and the further color change upon SDS addition highlights the specificity of the PEG–PCL NPs–Bradford reagent system for SDS detection. These observations suggest that the designed nanoparticles enhance the sensitivity of SDS detection and provide a clear and distinct visual indication of SDS presence, making the system highly effective for rapid and onsite environmental monitoring. This selective detection capability, combined with the ease of visual inspection and quantification, underscores the potential of PEG–PCL nanoparticles in developing sensitive and specific methods for environmental and industrial applications.

### Selectivity assessment and quantitative determination of SDS using PEG–PCL NPs

**Figure 6:**
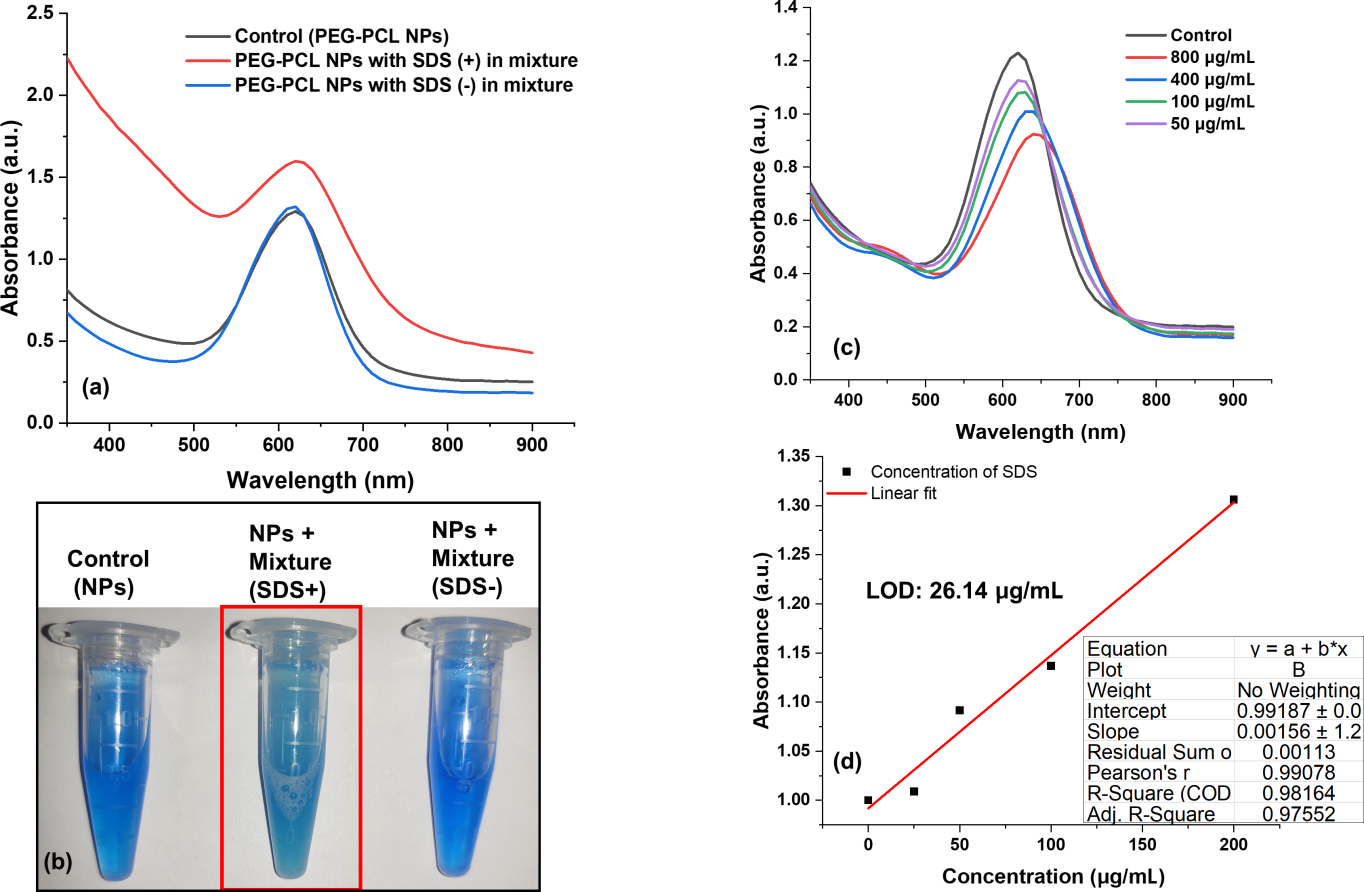
a) Spectrophotometric and b) colorimetric (marked with red) detection of SDS in a mixture of different metal ions and surfactants using PEG–PCL NPs. c) Plasmonic shift upon addition of different concentrations of SDS, and d) linear equation for quantification of the SDS detection limit.

To comprehensively evaluate the specificity of the PEG–PCL nanoparticle system towards SDS detection, further experiments were performed to observe color changes and the absorption spectra of NPs in the presence and absence of SDS and other metals and surfactants. This investigation is essential because heavy metal ions often coexist with surfactants in environmental matrices, which could potentially interfere with the detection system accuracy. To simulate these conditions, heavy metal ions (such as Al^3+^, Cd^2+^, Zn^2+^, Hg^2+^, Ni^2+^, Cu^2+^, Cr^3+^, Pb^2+^, Fe^3+^, Co^2+^, and As^3+^) and surfactants (such as CTAB, Tween 20, and Triton X-100) were introduced at concentrations of 1 ppm and 0.1%, respectively. These were mixed with the PEG–PCL NPs–Bradford reagent detection probe in a final volume of 1 mL. In the presence of SDS along with other metal ions/surfactants, significant color changes from blue to light blue and plasmonic shifts in the NPs were observed, as documented in Figure 6a,b.

These changes indicate a successful interaction between SDS and the PEG–PCL NPs–Bradford reagent complex, confirming the presence of SDS. Conversely, in the absence of SDS, there were no significant changes in absorbance or color, consistent across all tested metal ions and surfactants, as well as in control samples with no added metals or surfactants. This lack of response in the absence of SDS highlights the specificity of the detection system, as it demonstrates that the PEG–PCL NPs do not respond to other potential contaminants or interfere with the detection of SDS. These findings confirm that the PEG–PCL nanoparticle-based system selectively and specifically detects SDS, even in complex environments containing various metals and surfactants.

After establishing the qualitative detection capability, the sensing probe was further evaluated for its ability to quantitatively measure SDS concentrations. The limit of detection (LOD) and limit of quantification (LOQ) were determined through a linear calibration plot, wherein varying concentrations of SDS were mixed with PEG–PCL NPs. As shown in Figure 6c, the plot demonstrated excellent linearity, with a high correlation coefficient (*R*^2^) value of 0.98. This high R^2^ value indicates a strong linear relationship between the absorbance measured by the spectrophotometer and the concentration of SDS, thus ensuring accurate and reliable quantification. The LOD and LOQ were calculated to be 26.14 and 79.23 µg/mL, respectively (Figure 6d). These values indicate the sensitivity of the detection system, with the ability to detect even low concentrations of SDS, making it suitable for environmental monitoring applications where low contamination levels must be detected. Furthermore, the performance of the PEG–PCL NPs-based detection system was compared with other nanoparticle-based detection methods for SDS, as summarized in Table 1. This comparison highlights the advantages of the present work, including its high specificity, rapid response time, and ability to effectively operate in complex matrices containing potential interferents, establishing its utility as a practical tool for environmental and industrial applications.

**Table 1:** List of previous works regading the detection of SDS using the nanoparticle system.

S.No	Nanoparticle-based system	Mode of detection	Limit of Detection	Ref.

1.	Gold nanocluster capped with polydiallyl dimethylammonium chloride	fluorescent	0.02 μg/mL	[18]
2.	Polyethyleneimine- and ascorbic acid-based nanoparticles	fluorescent	0.051 μg/mL	[27]
3.	Carbon nanoparticles using 5-hydroxytryptamine	fluorescent	2.5 nM	[38]
4.	Polyelectrolyte microcapsules	fluorescent	10–50 μg/mL	[39]
5.	PEG–PCL nanoparticles	colorimetric and spectrophotometric	26.14 μg/mL	this work

## Conclusion

This study introduces an optimized colorimetric detection method for SDS using PEG–PCL NPs combined with the Bradford reagent. The PEG–PCL nanoparticles were synthesized using the ring-opening copolymerization method. Further physicochemical characterization revealed that synthesized nanoparticles were quasi-spherical and had a negative surface charge. To make a colorimetric/spectrophotometric sensor, PEG–PCL NPs and the Bradford reagent were mixed in a fixed ratio. Notably, the method provides a broad detection range and a low detection limit for SDS, demonstrating significant sensitivity and selectivity. Specifically, the method effectively detects SDS concentrations across a wide range (0–200 µg/mL) with a detection limit of 26.14 µg/mL. This allows the method to be versatile in quantifying SDS from trace amounts to higher concentrations, such as those typically found in different products involved in environmental contaminants. Moreover, the optimized approach shows exceptional selectivity for SDS even in the presence of potential interfering substances, including heavy metals, cationic surfactants, and nonionic surfactants. This high selectivity is crucial for accurate detection in complex matrices. The ability to detect SDS without significant interference from other substances underscores the method suitability for field-based testing and routine monitoring of SDS contamination in various sources. The developed method offers several key advantages: it is simple, rapid, cost-effective, and it has high stability. These findings highlight the potential of this method for SDS detection from different sources, providing a valuable tool for routine surveillance and assessment.

## Data Availability

All data that supports the findings of this study is available in the published article and/or the supporting information of this article.
